# Ship Radiated Noise Recognition Technology Based on ML-DS Decision Fusion

**DOI:** 10.1155/2021/8901565

**Published:** 2021-10-07

**Authors:** Biao Wang, Chengxi Wu, Yunan Zhu, Mingliang Zhang, Hanqiong Li, Wei Zhang

**Affiliations:** School of Electronic Information, Jiangsu University of Science and Technology, Zhenjiang 212100, China

## Abstract

Ship radiated noise is an important information source of underwater acoustic targets, and it is of great significance to the identification and classification of ship targets. However, there are a lot of interference noises in the water, which leads to the reduction of the model recognition rate. Therefore, the recognition results of radiated noise targets are severely affected. This paper proposes a machine learning Dempster–Shafer (ML-DS) decision fusion method. The algorithm combines the recognition results of machine learning and deep learning. It uses evidence-based decision-making theory to realize feature fusion under different neural network classifiers and improve the accuracy of judgment. First, deep learning algorithms are used to classify two-dimensional spectrogram features and one-dimensional amplitude features extracted from CNN and LSTM networks. The machine learning algorithm SVM is used to classify the chromaticity characteristics of radiated noise. Then, according to the classification results of different classifiers, a basic probability assignment model (BPA) was designed to fuse the recognition results of the classifiers. Finally, according to the classification characteristics of machine learning and deep learning, combined with the decision-making of D-S evidence theory of different times, the decision-making fusion of radiated noise is realized. The results of the experiment show that the two fusions of deep learning combined with one fusion of machine learning can significantly improve the recognition results of low signal-to-noise ratio (SNR) datasets. The lowest fusion recognition result can reach 76.01%, and the average fusion recognition rate can reach 94.92%. Compared with the traditional single feature recognition algorithm, the recognition accuracy is greatly improved. Compared with the traditional one-step fusion algorithm, it can effectively integrate the recognition results of heterogeneous data and heterogeneous networks. The identification method based on ML-DS proposed in this paper can be applied in the field of ship radiated noise identification.

## 1. Introduction

Radiated noise is an important source of information for underwater acoustic target recognition. In recent years, research on radiated noise has been a hot topic. Many countries have launched research on this, which has important guiding significance for national security, shipping, and traffic management and marine ecological protection [1, 2]. Radiated noise usually consists of mechanical noise, propeller propulsion noise, engine noise, and so on. It has the characteristics of low frequency, strong interference, and high noise. These features can characterize the invariable physical characteristics and space orientation information of ships [3–6]. Therefore, improving the recognition accuracy and efficiency has attracted widespread attention in ship target recognition [7].

Currently, target recognition of radiated noise includes two modules: feature extraction and classification recognition. Feature extraction is the process of extracting various features from radiated noise signals. MFCC [8–10], wavelet feature [11–13], and Hilbert Huang feature [14–16] are often used in traditional radiated noise feature extraction. How to extract features and what features to extract suitable for the recognition and classification of radiated noise has always been a topic of research by researchers. The system needs to store and process data under limited resource conditions. Reducing computing costs and communication costs are also issues that often need to be considered in the identification process [17–19]. However, different environments are suitable for extracting different features; the background of this paper is to use traditional features with a relatively low recognition rate and deep learning to extract features to improve the recognition effect of radiated noise under different environmental backgrounds.

Mel cepstrum coefficient MFCC and energy are the traditional way to extract the audio features of radiated noise. Information is extracted from the Mel filter bank and becomes the basis of most speech recognition technologies. It is currently widely used in the field of radiated noise recognition. In [7], Zhen first proved that the MFCC feature indicates that the underwater acoustic signal is effective. In [20], Cheng et al. used machine learning algorithms to find that the DBN method has the best MFCC feature recognition performance for passive sonar targets. Because of its excellent stability and high recognition rate, this paper considers using traditional Chroma_STFT features with a relatively low recognition rate as research to improve the recognition accuracy after fusion. Chromaticity feature is the collective name of chroma vector and chromagram. The chroma vector is a vector containing 12 elements. Each of these elements represents the energy at 12 pitch levels over time, such as a frame. The energy of the same pitch level with different octaves will accumulate, and the chromaticity map is a sequence of chromaticity vectors [21].

Recognition is the process of cognition of unknown signals, which requires a certain degree of training to achieve the effect. Therefore, traditional feature extraction methods are usually combined with traditional classifiers for classification and recognition. The extracted signal features are recognized in decision trees, clustering, and SVM models, which have good results. In [22], Jiang et al. proposed that multiscale spectral feature extraction features can effectively improve the accuracy of dynamic target recognition. In [23], Xie et al. proposed a novel method that uses improved variational mode decomposition (IVMD), normalized maximum information coefficient, and permutation entropy (PE) based on particle swarm optimization. The classification is implemented in the support vector machine multiclassifier.

With the development of deep learning in the field of underwater acoustics, deep learning algorithms are often used for the recognition and exploration of underwater acoustic targets. In [24], Jiang et al. used the CNN network to detect and classify the whistle of killer whales and albacore pilot whales and achieved good recognition results. In [25], Jin et al. used the GAN network to extract features of LOFAR, which effectively improved the recognition effect of underwater acoustic targets. In [26], Ibrahim et al. used DNN to extract features in the sound to identify grouper species, and their results were significantly better than earlier methods. Compared with the machine learning model, the deep learning algorithm can greatly improve the recognition accuracy of the model. At the same time, due to the strong robustness of DNN, it can recognize underwater acoustic signals under noisy conditions.

This paper proposes an improved feature fusion ML-DS algorithm based on the fusion of different features in the same feature extraction method proposed by Zhang et al. [27]. It combines the features of multidimensional and multiclassifier fusion to identify radiated noise targets, solves the limitation of single feature recognition accuracy, and combines the results of deep learning and machine learning.

The rest of the paper is organized as follows: Section 2 introduces the network for feature extraction and recognition, Section 3 discusses the multifeature ML-DS decision fusion algorithm, and Section 4 introduces the experimental results and analysis. Finally, Section 5 summarizes this work.

## 2. Feature Extraction

This method uses the chromaticity features of short-time Fourier transform (STFT) in the traditional method to obtain the classification result on the machine learning classifier. In the deep learning method, the CNN network is used to extract the two-dimensional LOFAR image features and classify them to obtain the recognition results. The LSTM network is used to extract the continuous amplitude feature of the signal and classify it to obtain the recognition result. The recognition and classification results of machine learning methods and deep learning methods are designed into the BPA of the fusion model. The fusion of different classifiers is realized through two D-S evidence theories at the decision level. Finally, the fusion of multidimensional features and multiclassifiers is realized, which effectively improves the recognition accuracy. At the same time, it can effectively reduce the time of complex feature fusion feature extraction and improve the recognition performance. Add −20 dB, −10 dB, 0 dB, and 10 dB to the dataset containing the radiation noise of four types of 9 ships of merchant ships, cargo ships, fishing vessels, and oil tankers to construct a dataset containing noise. The recognition results show that the method has a good recognition effect and has important theoretical and practical value.

### 2.1. CNN Extracts Image Features

**Figure 1:**
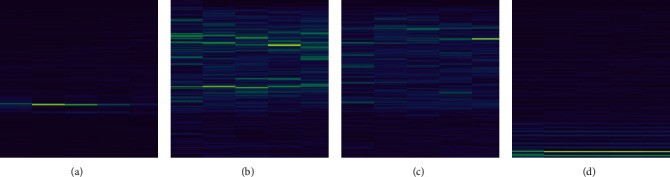
LOFAR spectrum image sample of ship radiated noise. (a) The LOFAR spectrum of a cargo ship. (b) The LOFAR spectrum of a cruise ship. (c) The LOFAR spectrum of a merchant ship. (d) The LOFAR spectrum of a fishing boat.

The LOFAR spectrum is the continuous time domain sampling of the underwater acoustic target signal and the time-varying information obtained by STFT. It is projected onto the time and frequency plane to form a three-dimensional map. It is usually used in the field of underwater acoustic target recognition [25, 28–30]. The task of tracking the target is accomplished by identifying the line features of the LOFAR spectrum image.  Figure 1 shows an example of a LOFAR spectrum image sample of ship radiated noise.

First of all, this article preprocesses the original radiated noise signals and estimates these signals to get a better spectrum. Secondly, when using the LOFAR spectrum, the original audio file is converted into a spectrum file through the STFT operation. The analysis window function of STFT makes it stable in different finite time intervals, so as to calculate different power spectra in a time [28]:

(1) $$\begin{array}{c} S\left(\omega ,\tau \right)=\int_{R}f\left(t\right)g\left(\bar{\omega }-\tau \right)e^{-jwt}\text{d}t, \end{array}$$

where *f*(*t*) is the signal entering the analysis, *e*^−*jwt*^ is the frequency limit effect, and *τ* is the time limit effect.

Finally, according to the intensity of the color, the energy in the frequency band is judged, and the attributes of the key feature energy lines of LOFAR are emphasized. The convolutional neural network is used to classify the frequency energy line features.

**Figure 2:**
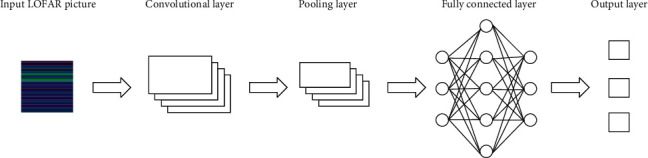
Schematic diagram of convolutional neural network structure.

The CNN model is usually used to classify image features. It is one of the most popular and widely used models in deep learning in recent years. It is possible to obtain effective representations directly from the original data through the alternate use of the convolutional layer and the pooling layer, automatically extract the local features of the image, and establish a dense and complete feature vector [31]. This paper studies the use of fusion technology to fuse different types of feature extraction, and the fusion of different classifiers uses CNN to extract the LOFAR image features of radiation noise, classifies and recognizes the LOFAR image features, and then determines the fusion of the recognition results.  Figure 2 shows a schematic diagram of the convolutional neural network structure.

The convolutional layer is composed of multiple feature maps. The convolutional layer performs convolution operation with a certain size and the original input image through the convolution kernel. It obtains the feature map of the next layer after the activation function. Each neuron in the feature map of the convolutional layer is connected to the local area of the feature map of the previous layer through a set of weights, and the pixel weighted summation is performed. The locally weighted sum is passed to a nonlinear activation function to obtain the value of each neuron in the stack. The calculation formula of the feature is as follows:

(2) $$\begin{array}{c} S_{j}^{l}=f\left(\sum_{i\in M_{j}}S_{j}^{l-1}*Q_{ij}^{l}+b_{j}^{l}\right), \end{array}$$

where *S*_*j*_^*l*^ represents *j* feature map of the *l* layer, *f*(·) represents the activation function, *S*_*j*_^*l*−1^ represents *j* feature map of *l* − 1 layer, *∗* represents the convolution operation, *Q*_*ij*_^*l*^ is the convolution kernel, and *b*_*j*_^*l*^ represents the bias.

Since the convolution operation has a linear relationship between the input matrix and the convolution kernel matrix, the activation layer must perform nonlinear mapping on them. The activation layer can solve problems that cannot be solved by the linear model. A nonlinear activation function is nested based on the output result of the convolution layer to activate the features extracted by the convolution layer. The network structure of this paper adopts the ReLU activation function. Compared with the Sigmoid and Tanh functions, it has faster convergence speed and alleviation of the gradient disappearance problem.

The pooling layer is used to extract the most representative features in the region, which can effectively reduce the size of the output feature map, reduce the calculation amount of the network model, and improve the accuracy of the network's feature extraction of the input image. The common pooling method usually extracts the maximum or average pixel value of the area as the value of the neuron in the pooling layer. All networks in this article use the maximum pooling method.

The fully connected layer is located after the convolutional layer and the pooling layer and summarizes the extracted features. It connects neurons with all neurons in the previous layer and integrates the features extracted from the convolutional layer or the pooling layer. Finally, it is connected with the output layer to return the classification result.

With the rapid development of CNN, it has a good effect and fast speed in extracting target features. It is gradually replacing the target detection method based on manual features and becoming the mainstream in the current target recognition and detection field.

### 2.2. LSTM Extracts Signal Features

**Figure 3:**
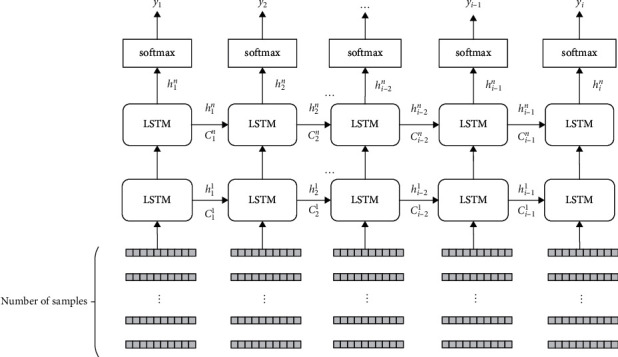
LSTM operating principle diagram.

The STM model is often used in the field of audio recognition. It has a better recognition and processing effect on time series signals. It can store long-term information, which can prevent the training process from being disturbed by the outside world. The LSTM model is composed of a series of timing modules, generally, including input gates, forget gates, and output gates. The gate control mechanism is used to control the flow of information in the memory block so that it has long-term and short-term memory capabilities [32–34].  Figure 3 shows the structure diagram of the operating principle of LSTM.

Assuming that *x* is the input data of the timing signal, the forward propagation formula can be expressed as [35]

(3) $$\begin{array}{c} I_{\text{t}}=\sigma \left(W_{I}\cdot \left[x_{t},\;h_{t-1},\;c_{t-1}\right]+b_{I}\right),\\ F_{t}=\sigma \left(W_{F}\cdot \left[x_{t},\;h_{t-1},\;c_{t-1}\right]+b_{F}\right),\\ C_{t}=F_{t}h_{t-i}+i_{t}\tanh\left(W_{C}\left[h_{t-1},\;C_{t-1}\right]+b_{C}\right),\\ O_{t}=\sigma \left(W_{O}\cdot \left[x_{t},\;h_{t-1},\;c_{t-1}\right]+b_{O}\right),\\ h_{t}=O_{t}\tanh\;C_{t}, \end{array}$$

where **I**_*t*_ is the input gate, **F**_*t*_ is the forget gate, *C*_*t*_ is the state of the cell unit after passing the input gate and the forget gate at all times, *O*_*t*_ is the cell state of the output gate, **h**_*t*_ is the output state of all LSTM units, and *σ*(*·*) is the activation function of sigmoid, **W**_*I*_, **W**_*F*_, **W**_*C*_,  and **W**_*O*_ are the LSTM implicit state weight matrices, and **b**_*I*_, **b**_*F*_, **b**_*C*_,  and **b**_*O*_ are the offset.

LSTM training can be divided into four steps: Step 1: forward propagation, calculating the network output value; Step 2: backward propagation, calculating the time and network error; Step 3: calculating the gradient value; and Step 4: updating the weight coefficient.

### 2.3. SVM Classifier Design

SVM is based on the traditional learning theory and the principle of structural risk minimization. It maps the nonlinear transformation to the high-dimensional space and linearly separates the samples in the high-dimensional feature space [36].

Assuming a hyperplane *w*^*T*^*x*+*b*=0 in a two-dimensional space, *P*(*x*_1_, *x*_2_,…, *x*_*n*_) is a point in the sample, and *x*_*i*_ is the *i* feature variable, then the distance from the point to the hyperplane is

(4) $$\begin{array}{c} d=\frac{\left|w_{1}\cdot x_{1}+w_{2}\cdot x_{2}+\cdots +w_{n}\cdot x_{n}+b\right|}{\sqrt{w_{1}{}^{2}+w_{2}{}^{2}+\cdots +w_{n}{}^{2}}}=\frac{\left|W^{T}\cdot X+b\right|}{\left\|W\right\|}. \end{array}$$

Among them, ‖*W*‖ is the norm of the hyperplane. If the hyperplane is determined, all the support vectors are found, and then the interval margin is calculated. Finally, the hyperplane corresponding to the largest value among all the margins is found.

It is necessary to determine *W* and *b* to maximize the margin, so the objective function of the optimization problem can be written as argmax_*w*,*b*_(min(*y*(*w*^*T*^ · *x*+*b*))(1/‖*w*‖)). Since *w* and *b* are enlarged in proportion, the result of *d* remains unchanged, so it can be simplified to

(5) $$\begin{array}{c} \left\{\begin{array}{c} \arg\max\left(\frac{1}{\left\|w\right\|}\right),\\ \text{s.t. }y\left(w^{T}\cdot x+b\right)-1\geq 0. \end{array}\right) \end{array}$$

Replace min(1/2)‖*w*‖^2^ with the objective function equivalent. It is transformed into a constrained optimization problem and solved by the Lagrangian multiplier method.

(6) $$\begin{array}{c} L\left(w,b,\alpha \right)=\frac{1}{2}\cdot \left\|w\right\|-\sum_{i=1}^{n}a_{i}\left(y_{i}\left(w\cdot x+b\right)-1\right). \end{array}$$

Find the partial derivative of *L* to get

(7) $$\begin{array}{c} \left\{\begin{array}{c} \frac{\partial L\left(w,b,\alpha \right)}{\partial w}=0\Rightarrow w=\overset{n}{\underset{i=1}{\sum }}a_{i}y_{i}x_{i},\\ \frac{\partial L\left(w,b,\alpha \right)}{\partial b}=0\Rightarrow \overset{n}{\underset{i=1}{\sum }}a_{i}y_{i}=0, \end{array}\right) \end{array}$$

and simplify to get

(8) $$\begin{array}{c} L\left(\alpha \right)=\frac{1}{2}\overset{n}{\underset{i=1}{\sum }}a_{i}a_{j}y_{i}y_{j}x_{i}^{T}x_{j}-\overset{n}{\underset{i,j=1}{\sum }}a_{i}a_{j}y_{i}y_{j}x_{i}^{T}x_{j}-b\overset{n}{\underset{i,j=1}{\sum }}a_{i}y_{i}+\overset{n}{\underset{i=1}{\sum }}a_{i}\\ =\overset{n}{\underset{i=1}{\sum }}a_{i}-\frac{1}{2}\overset{n}{\underset{i,j=1}{\sum }}a_{i}a_{j}y_{i}y_{j}x_{i}^{T}x_{j}. \end{array}$$

Therefore, it is finally reduced to the objective function:

(9) $$\begin{array}{c} \max L\left(a\right)=\overset{n}{\underset{i=1}{\sum }}a_{i}-\frac{1}{2}\overset{n}{\underset{i,j=1}{\sum }}a_{i}a_{j}y_{i}y_{j}x_{i}^{T}x_{j},\\ \text{s.t. }\left\{\begin{array}{c} \overset{n}{\underset{i=1}{\sum }}a_{i}y_{i}=0,\\ a_{i}>0,\;i=1,2,\ldots ,n, \end{array}\right) \end{array}$$

This paper adopts a linear kernel *κ*(*x*, *y*)=*x*^*T*^*y* and finds all support vectors *a* to determine *w*, *b*. Then, by calculating the distance from the data point to the hyperplane, the category of the characteristic data point is determined.

### 2.4. Classifier Recognition Result

#### 2.4.1. The Recognition Rate of LOFAR Spectrogram in CNN

We add −20 dB, −10 dB, 0 dB, and 10 dB noise to the original radiated noise and then use the STFT algorithm to extract the characteristics of the LOFAR spectrum of the radiation noise and save the image. Due to the limited computer hardware requirements, it is necessary to zoom and crop the image size of the LOFAR spectrogram, save it as a 32 *∗* 32 size image, and use the CNN network to extract the features and recognize and classify the LOFAR spectrogram. The CNN network is designed as a two-layer convolutional layer, including 64 convolution kernels with a size of 3 *∗* 3, a pooling layer and a fully connected layer of 512 elements, and outputs the judgment results of four types of ships. Set the learning rate to 0.0001, the loss function uses the cross-entropy loss function, and the optimizer selects Adam. After 100 iterations, the results of four sets of training models are finally obtained. The recognition accuracy of the model with 10 dB radiated noise can reach 97.27%.  Table 1 shows the final recognition results.

#### 2.4.2. Recognition Rate of Radiated Noise Signal in LSTM

We add −20 dB, −10 dB, 0 dB, and 10 dB noise to the original radiated noise. LSTM has good characteristics in recognizing time series signals by extracting the original data amplitude characteristic data and identifying classification. In the experiment, the structure of the input feature data is changed to 49 dimensions; 45 steps are set and finally put into the classifier model for training and testing. The model chooses a 1-layer 64 LSTM unit and a 2-layer 32-unit LSTM network structure to achieve the optimal situation. After 60 iterations, the final loss of the experimental results tends to be flat, and the results of four sets of training models under different SNRs are obtained. The recognition accuracy of the model with 10 dB radiated noise can reach 95.68%. The final recognition results are shown in Table 2.

#### 2.4.3. Chroma_STFT Feature Recognition Rate in SVM

**Figure 4:**
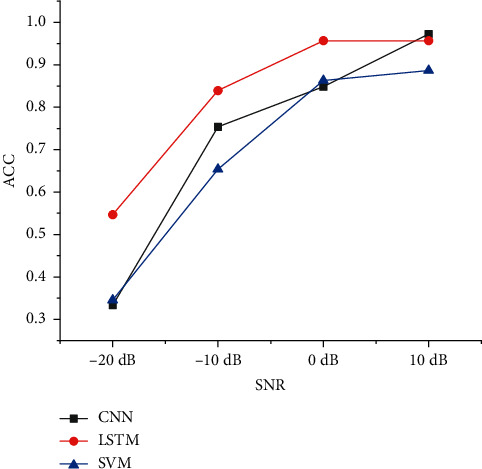
Classification and recognition accuracy of different models under different SNR conditions.

We add −20 dB, −10 dB, 0 dB, and 10 dB noise to the original radiated noise. Then, we use the Librosa toolbox to extract the chromaticity features (Chroma_STFT) of the original signal and reshape the feature array structure to match the input structure of SVM. Finally, the extracted features are put into the SVM classifier to classify the features because the experiment considers the fusion of the recognition results of classifiers with relatively low recognition rate to improve the overall recognition effect. Therefore, SVM selects a linear kernel function with a relatively low recognition rate to recognize and classify features. The recognition accuracy of the model with 10 dB radiated noise can reach 88.64%.  Table 3 shows the final recognition results.  Figure 4 shows the classification and recognition accuracy of different models under different SNR conditions.

## 3. Multifeature ML-DS Decision Fusion Algorithm

**Figure 5:**
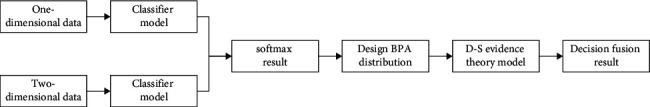
Flowchart of decision fusion of different types of data.

Decision fusion is a process of making secondary judgments on the recognition results of the classifier. Researchers often use the D-S evidence theory in the multisensor fusion theory to fuse the recognition results. It is a mathematical algorithm with uncertain reasoning and has weaker conditions than other methods to directly express the ability of information conflicts [37].  Figure 5 shows a flowchart of decision fusion of different types of data.

### 3.1. Design of Fitness Function and BPA

According to the research background of this article, the abstract recognition framework is *θ*={*A*, *B*, *C*, *D*}. In practice, there is no need to fully consider all combinations of *θ*, so this article only considers the classification of four probability models.

The basic probability distribution function is *m*{*A*},  *m*{*B*},  *m*{*C*},  *m*{*D*}. According to the D-S theory, the mass function under the current recognition framework can be expressed as *m*(∅)=0 and ∑_*A*⊆*θ*_*m*(*A*)=1.

According to the above formula, to obtain the synthesis rule *m*_1234_, we first obtain the value of the normalization coefficient 1 − *K*. Based on the research background of this article, there is only one target to be identified, so the intersections of *A*, *B*, *C*, and *D* are all empty:

The first step is to calculate the conflict factor *K*:

(10) $$\begin{array}{c} 1-K=\sum_{A\cap B\cap C\cap D\neq \emptyset }m_{1}\cdot m_{2}\cdot m_{3}\cdot m_{4}\\ =\sum_{A\cap B\cap C\cap D\neq \emptyset }m_{1}\left(A\right)\cdot m_{2}\left(B\right)\cdot m_{3}\left(C\right)\cdot m_{4}\left(D\right). \end{array}$$

The second step is to calculate the combined BPA of the four types of ship identification results according to the evidence rules.

The mass function value of *A* combination is given by

(11) $$\begin{array}{c} m_{1}⊕m_{2}⊕m_{3}⊕m_{4}\left(\left\{A\right\}\right)=\frac{1}{1-K}\sum_{A\cap B\cap C\cap D=\left\{A\right\}}m_{1}\cdot m_{2}\cdot m_{3}\cdot m_{4}\\ =\frac{1}{1-K}\cdot m_{1}\left(\left\{A\right\}\right)\cdot m_{2}\left(\left\{A\right\}\right)\cdot m_{3}\left(\left\{A\right\}\right)\cdot m_{4}\left(\left\{A\right\}\right). \end{array}$$

The mass function value of *B* combination is given by

(12) $$\begin{array}{c} m_{1}⊕m_{2}⊕m_{3}⊕m_{4}\left(\left\{B\right\}\right)=\frac{1}{1-K}\sum_{A\cap B\cap C\cap D=\left\{B\right\}}m_{1}\cdot m_{2}\cdot m_{3}\cdot m_{4}\\ =\frac{1}{1-K}\cdot m_{1}\left(\left\{B\right\}\right)\cdot m_{2}\left(\left\{B\right\}\right)\cdot m_{3}\left(\left\{B\right\}\right)\cdot m_{4}\left(\left\{B\right\}\right). \end{array}$$

The mass function value of *C* combination is given by

(13) $$\begin{array}{c} m_{1}⊕m_{2}⊕m_{3}⊕m_{4}\left(\left\{C\right\}\right)=\frac{1}{1-K}\sum_{A\cap B\cap C\cap D=\left\{C\right\}}m_{1}\cdot m_{2}\cdot m_{3}\cdot m_{4}\\ =\frac{1}{1-K}\cdot m_{1}\left(\left\{C\right\}\right)\cdot m_{2}\left(\left\{C\right\}\right)\cdot m_{3}\left(\left\{C\right\}\right)\cdot m_{4}\left(\left\{C\right\}\right). \end{array}$$

The mass function value of *D* combination is given by

(14) $$\begin{array}{c} m_{1}⊕m_{2}⊕m_{3}⊕m_{4}\left(\left\{D\right\}\right)=\frac{1}{1-K}\sum_{A\cap B\cap C\cap D=\left\{D\right\}}m_{1}\cdot m_{2}\cdot m_{3}\cdot m_{4}\\ =\frac{1}{1-K}\cdot m_{1}\left(\left\{D\right\}\right)\cdot m_{2}\left(\left\{D\right\}\right)\cdot m_{3}\left(\left\{D\right\}\right)\cdot m_{4}\left(\left\{D\right\}\right). \end{array}$$

Thus, the combined function *m*_1234_ is obtained. According to the mass function synthesized by Dempster, the reliability function and likelihood function of the combined mass function for each type of ship classification can also be calculated. *A*, *B*, *C*, and *D,* respectively, represent the current identification situation of four types of target ships under the tank.

In the third step, the model uses a combination of probability distribution functions to find the trust function and finds the likelihood function according to the degree of trust that the proposition is not false. Finally, it can find the probability of accurate decision fusion Table 4 shows abbreviations and notations of the symbols.

### 3.2. Specific Implementation Steps

**Figure 6:**
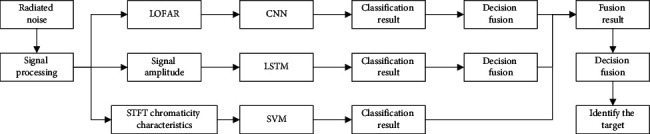
Dual decision-level fusion recognition framework based on evidence theory.

This paper focuses on the recognition and fusion of different types of ships under different dimensional feature conditions. The CNN network is used to extract the LOFAR spectrogram features of the two-dimensional image. The LSTM network is used to extract the features of the one-dimensional time domain signal amplitude, and the SVM classifier is used to extract STFT chromaticity features. The predicted results are fused for decision-making to fuse features of different dimensions and the recognition results of different classifiers. It can enrich the types of recognition, expand the range of fusion feature types, and provide new ideas for feature selection in underwater acoustic radiation noise recognition research. At the same time, the classification results of the low recognition rate model and the high recognition rate model can be merged to improve the recognition effect of the model and help the model improve the range of decision-making capabilities.  Figure 6 shows a dual decision-level fusion recognition framework based on evidence theory.

The specific steps to identify and classify radiated noise based on a decision-level fusion of different features under different classifier conditions are as follows.

The first step is to extract easily obtainable signal amplitude, LOFAR spectrum, Chroma_STFT, and other three characteristics to construct a dataset for the four types of ship targets *A*, *B*, *C*, and *D*.

In the second step, the CNN network is used to extract the LOFAR spectrogram features of the radiation noise for classification. The LSTM network is used to extract the signal amplitude features for classification, and the STFT chromaticity features are extracted from the original signal for classification in the SVM classifier.

The third step is to predict the probability of the category of the target noise from the four results of the classification and recognition of the three classification models. At the same time, a BPA model was constructed based on the predicted results.

The fourth step is to use D-S evidence theory to perform decision-level fusion on the prediction results of different feature categories to obtain decision fusion results under different classifier conditions. Among them, the machine learning method alone does not have a good fusion effect under the condition of a low SNR. Therefore, the average classification recognition probability is used to construct the BPA model, and it is no longer integrated separately.

The fifth step is to perform another decision fusion on the results of the decision fusion of different classifiers and fuse the learning results of the machine learning classifier to obtain the final decision fusion result. Finally, the fusion results of deep learning and machine learning are combined to improve the overall recognition effect of the recognition system.

Due to the fusion method of different classifiers, the conditions for feature selection and the BPA distribution are different. According to formulas (10)–(14), it can be seen that the results of different classifiers cannot be mixed and fused directly. Therefore, every five classification results are separately fused to make a decision and judgment. First, the results of one of the classifiers are mixed, and then, the recognition results of three different neural networks and classifiers are fused. Finally, the recognition accuracy rate after deep learning fusion is fused with the average recognition probability determined by the machine learning algorithm classifier to make the second decision fusion method. The experiment has realized the decision fusion result of the hybrid multiclassifier, which can better realize the effect of feature fusion.

## 4. Experiment Results and Analysis

This section shows the first decision fusion classification and recognition results of the CNN and LSTM models and the second decision fusion classification results of combing the three models. In order to verify the effectiveness of the proposed deep learning and machine learning decision fusion algorithm for radiated noise target recognition, the dataset is divided into four types of radiated noise from fishing vessels, merchant ships, oil tankers, and cargo ships. Each type of vessel contains 1800, 1200, 1800, and 1800 radiated noise audio files, corresponding to the radiated noise data of 9 ships. At the same time, −20 dB, −10 dB, 0 dB, and 10 dB noise are added to simulate a complex background noise environment to form a dataset under different SNR conditions. The training set is four times the test set.

Different dimensional features adopt different feature extraction methods, Therefore, the number of feature training times and prediction dimensions are also very different. In the experiment, the one-dimensional and two-dimensional features extracted from the 600, 900, and 1200 audio segments under the deep learning network are divided into five groups. The first decision fusion judgment is made. During the training, 480,720,960 radiated noise audio clips were fused with the LSTM and CNN networks. Then, the length of each type of ship timing sequence signal is retained in the LSTM and CNN network test set with 24, 36, and 48 fused judgment results, respectively. Finally, it is fused with the recognition effect discriminated by the SVM classifier, and the new BPA model is combined for the second fusion to achieve the best feature fusion effect.

**Figure 7:**
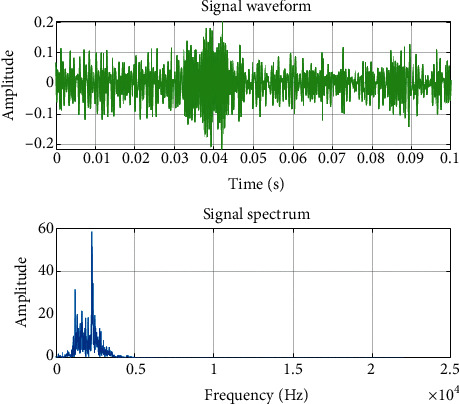
Waveform and frequency spectrum of the original audio.

In order to demonstrate the feature extraction method suitable for radiated noise, Table 5 shows the radiated noise audio information of the dataset, and Figure 7 shows part of the radiated noise data characteristics.

### 4.1. Convergence Comparison of CNN Network

**Figure 8:**
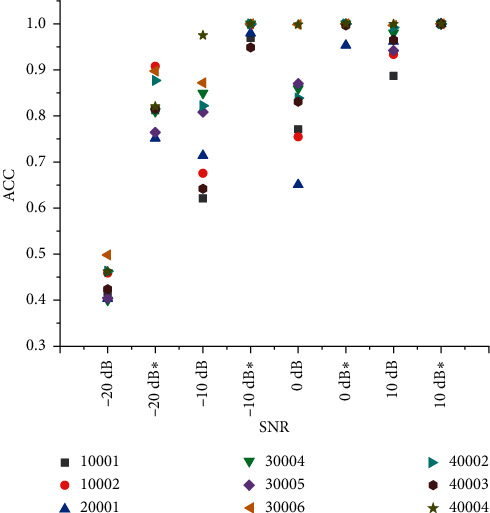
Accuracy comparison of CNN network before and after fusion under different SNRs (^*∗*^ is the result after fusion).

The CNN network is used to identify and classify the two-dimensional features of the LOFAR image extracted from the radiation noise. According to the fusion method of five parts, 24, 36, and 48 groups of fusion results are obtained from the test set which accounts for 20% of the dataset. We calculate the average recognition rate according to the fusion result. Finally, we get the recognition accuracy before fusion shown in Table 6 and the recognition accuracy after fusion shown in Table 7, and Figure 8 shows the comparison of the accuracy before and after fusion of the CNN network under different SNRs.

It can be seen from the table that the recognition rate of radiation noise with high SNR before fusion is higher. The recognition rate of low SNR radiated noise is generally low, with the lowest being only 40.03%. After the decision fusion of the CNN recognition results, the high SNR is close to 100%. The recognition rate of low SNR is nearly doubled. The lowest increase was also 34.79%. It is concluded that in different SNR datasets, the fusion under a single feature has a better effect on high SNR recognition. The recognition of low SNR has also been significantly improved, but the improvement effect is limited, and some fusion results do not exceed 90%.

It is concluded that the type of radiated noise can be effectively identified in the high SNR dataset, and the radiated noise can be misjudged in the low SNR dataset. However, after the decision-making fusion of the recognition of LOFAR images by the CNN network, the recognition effect has been significantly improved. In practical applications, collecting datasets of radiation noise under different working conditions will have a certain impact on the recognition results. Therefore, it is necessary to consider the design of the dataset in practical applications and fully consider the impact of the environment as much as possible and design models according to different datasets.

### 4.2. Convergence Comparison of LSTM Network

**Figure 9:**
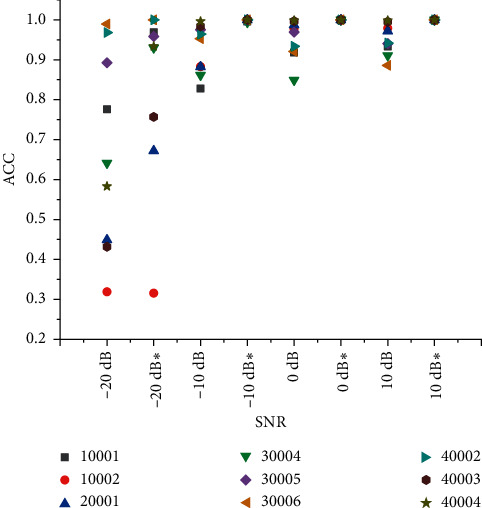
Comparison of accuracy of LSTM network before and after fusion under different SNRs (^*∗*^ is the result after fusion).

The LSTM network is used to identify and classify the characteristics of the waveform data extracted from the radiated noise. The fusion results of 24, 36, and 48 groups are obtained from the test set which accounts for 20% of the dataset according to the fusion method of five parts. We calculate the average recognition rate according to the fusion result. Finally, we get the recognition accuracy before fusion shown in Table 8 and the recognition accuracy after fusion shown in Table 9, and Figure 9 shows the comparison of the accuracy before and after fusion of the LSTM network under different SNRs.

It can be seen that the recognition rate of high SNR radiation noise is higher before fusion. The recognition rate of low SNR radiation noise is generally low, and the lowest is only 31.89%. After the decision fusion of the CNN recognition results, the high SNR is close to 100%. Except for the poor audio fusion result of 010002 radiated noise, the recognition rate of radiated noise of other ships has improved greatly. We analyze 36 sets of fusion results of the 010002 radiated noise audio fusion process. It is known that the recognition results of 010002 radiated noise and 020001 radiated noise in the −20 dB environment are relatively close, resulting in the misjudgment of the fusion algorithm. It is concluded that when the recognition rate of a single target is lower than a certain range, the fusion result is not ideal. From the analysis of the fusion recognition results of LSTM and CNN in the −20 dB environment, it can be seen that the recognition rate of a single network is less than 40%, and the recognition effect will be misjudged. With the expansion of ship types and datasets, the recognition effect will be better and better, and the result of fusion will be better.

### 4.3. SVM Recognition Results under Different SNRs

The SVM classifier is used to identify and classify the features of STFT chromaticity data extracted from radiated noise. The average result is identified from the test set that accounts for 20% of the dataset. Machine learning algorithms have lower recognition results under low SNR conditions. Machine learning algorithms alone cannot effectively participate in decision fusion. Therefore, SVM does not participate in the first decision fusion and only calculates the average recognition rate of each group of recognition results. The final decision fusion recognition result can be obtained after the fusion of the calculated recognition rate and the deep learning algorithm. First, we get the recognition of the four types of target radiated noise of different types of ships shown in Tables 10–13. *A*, *B*, *C*, and *D* correspond to their recognition rates, respectively.

**Figure 10:**
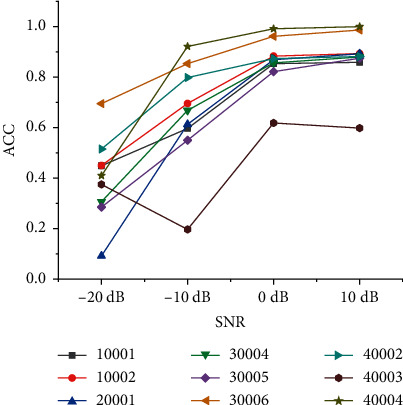
Comparison of the accuracy of SVM classifiers before fusion under different SNRs.

From the recognition result data, it is found that the recognition results of different types of ships under low SNR conditions are generally low. Among them, the recognition effect of Type *B* ships is the worst, and the chromaticity characteristics of Type *B* ships are not significantly different from other ships, resulting in a high probability of misjudgment of Type *A* ships. The recognition rate of Type *B* ships under high SNR conditions is higher. However, the recognition results of most types of ships do not exceed 90%, which is far lower than the recognition effect of deep learning algorithms under the same SNR dataset Figure 10 shows comparison of the accuracy of SVM classifiers before fusion under different SNRs.

### 4.4. The Final Fusion Result under Different Features and Classifier Conditions

After two fusions of deep learning algorithms and one fusion of machine learning algorithms, the final fusion experiment results are obtained.  Table 14 shows the recognition results of the final decision fusion under the −20 dB noise environment, and Table 15 shows the recognition results of the final decision fusion under the −10 dB noise environment. The results show that, under the condition of −20 dB, the recognition accuracy of radiated noise is more than 90% except that the recognition rate of class *B* ship radiated noise is lower than 80%, and the fusion identification data of some types of ship radiated noise are close to 100%. Compared with the traditional single feature and single classifier feature fusion, it has a significant improvement, which is helpful for multiangle decision-making of underwater acoustic targets.

In order to explore the situation where the fusion result of *B* type ship radiated noise recognition is significantly lower, from the fusion data of the three sets of classifiers analyzed in the first three sections, it can be seen that the recognition rate of SVM for 020001 ships under low SNR conditions is low, which is lower than the recognition results of deep learning algorithms. In the fusion process, the machine learning algorithm adopts the average recognition rate fusion judgment, which will have a certain influence on the fusion judgment. In response to this problem, the proposed solution is to modify the extracted features to improve the recognition and classification effect of a single classifier to improve the structure of the BPA, which can effectively improve the recognition accuracy.

The recognition rate of −10 dB ship radiated noise reaches 100%. Compared with the average recognition rate of CNN, LSTM, and SVM before a fusion, they have, respectively, increased by 22.45%, 7.48%, and 34.35%. Compared with the average recognition rate of CNN and LSTM after a fusion, they have, respectively, increased by 1.17% and 0.12%. It can effectively fuse the recognition results of different classifiers. Among them, the recognition accuracy of the STFT chromaticity feature of the 040003 ships under the SVM model is only 19.67%, indicating that this feature has a poor feature recognition effect on the 040003 ships, and the traditional machine learning algorithm is not ideal for the model recognition effect. However, the recognition rates of the same type of ship after the first fusion of CNN and LSTM models reached 94.88% and 100%. Then, it is fused with the judgment result of the SVM classifier with a lower recognition rate to obtain a nearly 100% recognition accuracy rate after decision-making. Therefore, it is proved that this method can effectively improve the recognition results of traditional machine learning algorithms under low SNR conditions, improve the accuracy of radiated noise recognition, and provide a new idea for the research of ship radiated noise recognition in complex environments.

At the same time, this experiment is based on the different characteristics of homologous signals to identify the characteristics of fusion. The two-dimensional image and one-dimensional signal features are, respectively, fused and judged. In the future, with the enrichment of underwater acoustic datasets, the model can also perform fusion experiments on the data of the same ship based on a simultaneous engraving of different data sources. For example, multiple data such as acoustic data, video data, and marine environmental data of the marine monitoring platform can identify and judge the collected radiated noise samples. We increase the fusion of different data sources to improve the accuracy of model recognition.

### 4.5. Future Work

This experiment adds noise with different SNRs based on the original ship noise to simulate the complex marine environment. Considering the real marine environment, it is also possible to combine ship radiated noise with underwater communication channels for further identification research. In the future, the integration of different types of data can be achieved through method improvements. A decision recognition algorithm that combines ship visual data recognition and radiated noise audio data recognition can realize the fusion of different data sources of the same ship to achieve better identification and judgment effect.

## 5. Conclusions

In this paper, the method of decision fusion is used to identify ship radiated noise. A fusion method of DS evidence decision theory for different dimensional characteristics is designed. The recognition results of machine learning and deep learning are used to extract the credibility of the results of different deep neural networks and classifiers. It designs a BPA function structure and adjusts the design of the mass function based on prior knowledge. Finally, the evidence decision theory is used to realize the feature fusion under different neural network classifiers, which effectively improves the recognition rate of ship radiated noise. (1) Radiated noise recognition technology is based on decision fusion, considering the fusion of recognition results from the decision-making level. It makes the fusion method more diverse and the recognition effect is better. (2) Compared with the recognition accuracy of using a single feature classifier, it uses the features of multiple signals to effectively improve the recognition accuracy after fusion. (3) Compared with the traditional one-time fusion algorithm, it uses the fusion algorithm proposed in this paper to effectively integrate the recognition results of heterogeneous data and heterogeneous networks. (4) After the first D-S evidence fusion of the deep learning model, the model can effectively identify the ship's radiation noise. The recognition accuracy rate under high SNR conditions is close to 100%, and the recognition accuracy rate under low SNR conditions is also greatly improved compared to traditional methods. The recognition result under the condition of low SNR after the secondary fusion of machine learning recognition results can be close to 100%, which improves the accuracy of decision-making fusion recognition under the condition of low SNR.

## Figures and Tables

**Table 1:** The recognition results of LOFAR features in the CNN network model test.

SNR	−20 dB	−10 dB	0 dB	10 dB
ACC	0.3333	0.7538	0.8485	0.9727

**Table 2:** Recognition results of signal amplitude characteristics in LSTM network model test.

SNR	−20 dB	−10 dB	0 dB	10 dB
ACC	0.547	0.8394	0.9568	0.9568

**Table 3:** Recognition results of STFT chromaticity features in SVM classifier.

SNR	−20 dB	−10 dB	0 dB	10 dB
ACC	0.3455	0.6538	0.8629	0.8864

**Table 4:** Abbreviations and notations.

Notations	Annotations
*S*_*j*_^*l*^	*j* feature map of the *l* layer
*f*(.)	The activation function
*Q*_*ij*_^*l*^	The convolution kernel
*b*_*j*_^*l*^	The bias
*O*_t_	The cell state of the output gate
**h**_*t*_	The output state of all LSTM units
*σ*(*g*)	The activation function of sigmoid
*x*_*i*_	The *i* feature variable
‖*W*‖	The norm of the hyperplane
*L*(*w*, *b*, *α*)	Lagrangian multiplier method
*θ*	The abstract recognition framework
*m*_1234_	Synthesis rule
*m*{*A*}	The basic probability distribution function
*K*	The conflict factor

**Table 5:** Dataset radiated noise audio information.

Type code	Ship type	Ship code	Ship details	Number of noise audio segments
*A*	Freighter	010001	Distance 83 chain, freighter	900
*A*	Freighter	010002	Distance 89 chain, freighter	900
*B*	Oil tanker	020001	Distance 29 chain, oil tanker	1200
*C*	Merchant ship	030004	Distance 50 chain, merchant shipping	600
*C*	Merchant ship	030005	Distance 83 chain, merchant shipping	600
*C*	Merchant ship	030006	Distance 30 chain, merchant shipping	600
*D*	Fishing boat	040002	Distance 28 chain, fishing boat	600
*D*	Fishing boat	040003	Distance 20 chain, fishing boat	600
*D*	Fishing boat	040004	Distance 23 chain, fishing boat	600

**Table 6:** CNN unfused recognition results.

Ship code	−20 dB	−10 dB	0 dB	10 dB
010001	0.4175	0.6211	0.7710	0.8869
010002	0.4589	0.6755	0.7547	0.9335
020001	0.4037	0.7139	0.6506	0.9617
030004	0.4003	0.8496	0.8592	0.9801
030005	0.4040	0.8085	0.8702	0.9420
030006	0.4982	0.8719	0.9984	0.9964
040002	0.4630	0.8223	0.8387	0.9919
040003	0.4240	0.6419	0.8310	0.9649
040004	0.4626	0.9751	0.9985	0.9997

**Table 7:** Recognition results after CNN fusion.

Ship code	−20 dB	−10 dB	0 dB	10 dB
010001	0.8123	0.9694	0.9996	1
010002	0.9078	0.9994	0.9993	1
020001	0.7516	0.9795	0.9533	1
030004	0.8098	0.9999	1	1
030005	0.7642	0.9981	1	1
030006	0.8974	1	1	1
040002	0.8770	1	1	1
040003	0.8155	0.9488	0.9965	1
040004	0.8207	1	1	1

**Table 8:** Recognition results before LSTM fusion.

Ship code	−20 dB	−10 dB	0 dB	10 dB
010001	0.7761	0.8280	0.9184	0.9332
010002	0.3189	0.8827	0.9777	0.9790
020001	0.4489	0.8824	0.9873	0.9717
030004	0.6415	0.8616	0.8491	0.9106
030005	0.8925	0.9752	0.9694	0.9406
030006	0.9895	0.9527	0.9207	0.8859
040002	0.9682	0.9644	0.9337	0.9420
040003	0.4318	0.9839	0.9969	0.9955
040004	0.5831	0.9963	0.9985	0.9986

**Table 9:** Recognition results after LSTM fusion.

Ship code	−20 dB	−10 dB	0 dB	10 dB
010001	0.9686	0.9958	1	0.9991
010002	0.3155	1	1	1
020001	0.6720	0.9999	1	1
030004	0.9302	0.9943	1	0.9999
030005	0.9583	1	1	1
030006	1	1	1	1
040002	1	1	1	1
040003	0.7569	1	1	1
040004	0.9336	1	1	1

**Table 10:** Recognition results of SVM classifier for four categories of target radiated noise under the condition of 10 dB noise.

Ship code	*A*	*B*	*C*	*D*
010001	**0.8589**	0.0467	0.0322	0.0622
010002	**0.8933**	0.0656	0	0.0411
020001	0.0992	**0.8925**	0.0033	0.005
030004	0.0117	0	**0.88**	0.1083
030005	0.0967	0.0133	**0.875**	0.015
030006	0	0	**0.9867**	0.0133
040002	0.0883	0.0033	0.0267	**0.8817**
040003	0.32	0.0517	0.03	**0.5983**
040004	0	0	0	**1**

Bold represents the recognition accuracy of a certain numbered ship under the correct classification category.

**Table 11:** Recognition results of SVM classifier for four categories of target radiated noise under the condition of 0 dB noise.

Ship code	*A*	*B*	*C*	*D*
010001	**0.8533**	0.05	0.0344	0.0622
010002	**0.8833**	0.0833	0.0022	0.0311
020001	0.1208	**0.8692**	0.0008	0.0092
030004	0.0217	0.0033	**0.8567**	0.1183
030005	0.15	0.0167	**0.8217**	0.0117
030006	0.0083	0	**0.9617**	0.03
040002	0.0867	0.0083	0.0317	**0.8733**
040003	0.27	0.0767	0.035	**0.6183**
040004	0.0067	0	0.0017	**0.9917**

Bold represents the recognition accuracy of a certain numbered ship under the correct classification category.

**Table 12:** Recognition results of SVM classifier for four categories of target radiated noise under the condition of −10 dB noise.

Ship code	*A*	*B*	*C*	*D*
010001	**0.5967**	0.14	0.1333	0.13
010002	**0.6956**	0.1833	0.0278	0.0933
020001	0.3333	**0.6133**	0.0325	0.0208
030004	0.0883	0.035	**0.6683**	0.2083
030005	0.2917	0.0967	**0.55**	0.0617
030006	0.055	0.0083	**0.8683**	0.0683
040002	0.1117	0.0167	0.0733	**0.7983**
040003	0.5233	0.1517	0.1283	**0.1967**
040004	0.04	0	0.0383	**0.9217**

Bold represents the recognition accuracy of a certain numbered ship under the correct classification category.

**Table 13:** Recognition results of SVM classifier for four categories of target radiated noise under the condition of −20 dB noise.

Ship code	*A*	*B*	*C*	*D*
010001	**0.4489**	0	0.2356	0.3156
010002	**0.45**	0	0.2211	0.3289
020001	0.685	**0.0917**	0.0375	0.1858
030004	0.36	0	**0.3067**	0.3333
030005	0.3967	0	**0.285**	0.3183
030006	0.1717	0	**0.695**	0.1333
040002	0.3367	0	0.1483	**0.515**
040003	0.365	0	0.26	**0.375**
040004	0.325	0	0.265	**0.41**

Bold represents the recognition accuracy of a certain numbered ship under the correct classification category.

**Table 14:** The final recognition results after two final decision-making fusions of evidence, in the −20 dB noise environment.

Ship code	*A*	*B*	*C*	*D*
010001	**0.9981**	0	0.0019	0
010002	**0.9522**	0	0	0.0478
020001	0.1970	**0.7601**	0	0.0429
030004	0.0200	0	**0.9800**	0
030005	0.0413	0	**0.9583**	0.0003
030006	0	0	**1**	0
040002	0	0	0	**1**
040003	0.0808	0	0	**0.9192**
040004	0.0251	0	0	**0.9748**

Bold represents the recognition accuracy of a certain numbered ship under the correct classification category.

**Table 15:** The final recognition results after two final decision-making fusions of evidence, under −10 dB noise environment.

Ship code	*A*	*B*	*C*	*D*
010001	**1**	0	0	0
010002	**1**	0	0	0
020001	0	**1**	0	0
030004	0	0	**1**	0
030005	0	0	**1**	0
030006	0	0	**1**	0
040002	0	0	0	**1**
040003	0	0	0	**0.9924**
040004	0	0	0	**1**

Bold represents the recognition accuracy of a certain numbered ship under the correct classification category.

## Data Availability

The data used to support the findings of this study are available from the corresponding author upon request.
